# The Development of the Radial Bow in Children: Normative Values

**DOI:** 10.3390/children12010101

**Published:** 2025-01-17

**Authors:** Samara F. Kass, Julia L. Conroy, Alisa Forsberg, Rebecca S. Li, Catherine C. May, Natasha S. McKibben, Julio J. Jauregui, Raymond W. Liu, Joshua M. Abzug

**Affiliations:** 1 Department of Orthopaedics, University of Maryland School of Medicine, Baltimore, MD 21201, USA; sfk40@case.edu (S.F.K.); jconroy@som.umaryland.edu (J.L.C.); aforsberg@som.umaryland.edu (A.F.); rebeccali@som.umaryland.edu (R.S.L.); catherine.may@som.umaryland.edu (C.C.M.); jabzug@som.umaryland.edu (J.M.A.); 2 Department of Orthopaedic Surgery and Rehabilitation, Oregon Health & Science University, Portland, OR 97239, USA; mckibbna@ohsu.edu; 3 Department of Pediatric Orthopaedics, Rainbow Babies and Children’s Hospital, Case Western Reserve University, Cleveland, OH 44106, USA; raymond.liu@uhhospitals.org

**Keywords:** adolescent, development, pediatric, radial bow, radiograph

## Abstract

Background: Radial bowing is necessary for forearm rotation. Fractures or deformities of the forearm that affect the radial bow may disrupt normal forearm rotation. Objective: The purpose of this study was to evaluate the development and establish normative values for the pediatric and adolescent radial bow. Methods: A retrospective review of radiographs from children aged 0–17 years with no previous history of an upper extremity fracture or condition was performed. Patient demographics, maximal radial bow location, and depth were recorded in millimeters and as percentages of the total radial length. The proximal, middle, and distal third radial bows were measured. Means and standard deviations were utilized to establish average measurements by age group. Pearson correlation coefficients assessed the relationship between radial bowing and age. Results: A total of 505 forearm radiographs were measured. The average age of the patients was 10.0 years (SD: 5.2). The maximal radial bow location remained consistent with age, with a mean value of 61.7%. The maximal radial bow depth marginally decreased with age, with a mean value of 7.7%. The proximal, middle, and distal third maximal radial bows (measured in millimeters) increased with age. Conclusions: The maximal radial bow depth marginally decreases with increasing age, while the location of the maximal radial bow remains consistent as age increases. These measurements may be used to assess fracture reduction to ensure proper restoration of the radial bow during fracture and congenital condition treatment. Furthermore, they can aid in the detection of subtle fractures of the radius, including plastic deformations and greenstick fractures.

## 1. Introduction

Radial bowing is necessary to permit the normal range of motion of the forearm. The radial bow, also known as the lateral curve, allows for pronation and supination of the forearm, in which the radius rotates around the stationary ulna [1,2]. Forearm fractures are extremely common in the pediatric and adolescent populations, accounting for approximately 25% of all pediatric fractures [3]. Furthermore, forearm fractures and/or plastic deformations of the forearm may disrupt the normal radial bow [2]. Congenital conditions may also lead to abnormal development of the radial bow.

Among the adult population, restoration of the radial bow during surgical intervention is important to maintain forearm range of motion as well as grip and pinch strength [1,4,5]. Schemitsch and Richards developed a technique for measuring the radial bow among a cohort of 55 adult patients that underwent open reduction and plate and screw fixation of both bone forearm fractures, which has been replicated numerous times in other studies [1,2,4]. In their study, the injured forearm was compared to the uninjured, contralateral side. Eighty percent of normal forearm rotation and grip strength could be expected in the injured arm when the location of the maximum radial bow was within four to five percent of the uninjured forearm [4]. These findings highlight the importance of establishing normative pediatric measurements of the radial bow, specifically when assessing the quality of a closed reduction or operative management [4].

Recent forearm fracture studies have included maximal radial bow depth and location as postoperative outcomes of interest within the pediatric and adolescent populations [6,7,8,9]. These investigations have utilized values established by Firl and Wunsch [2] as well as contralateral and control group comparisons to evaluate the restoration of the radial bow following operative management of pediatric and adolescent forearm fractures.

A recent study conducted by Nedder et al. investigated the development of the radial bow using computed tomography (CT) images [10]. This 3-dimensional investigation concluded that the normalized maximal, distal third, and middle third radial bows increased with age [10]. However, the normalized location of the radial bow and proximal third of the radial bow remain constant with increasing age [10]. These results suggest that the proportional shape of the radius changes throughout development, although there seems to be a qualitative plateau at 8 years of age. That said, CT images are not often utilized to evaluate acute pediatric and adolescent forearm fractures. Thus, a plain radiographic investigation of the radial bow development is warranted to inform clinical practice.

While maximum radial bowing has been utilized as a postoperative outcome measure, normative radiographic values of radial bow location and depth have yet to be reported in the pediatric and adolescent populations. The purpose of this study was to assess the development of the radial bow using plain radiographs to establish normative values of the radial bow among the pediatric and adolescent populations.

## 2. Materials and Methods

Following Institutional Review Board approval, a retrospective review of radiographs from children aged 0–17 years with no previous history of upper extremity injury or associated conditions was performed. Radiographs were obtained from medical charts of patients that underwent forearm radiographs as part of routine care by the Department of Pediatric Orthopaedics at the University of Maryland Medical Center located in Baltimore, Maryland, USA. This institution serves the diverse population of Maryland, which is made up of various racial/ethnic groups, including White (48.3%), Black or African American (31.0%), Hispanic (10.6%), Asian (7.0%), two or more races/ethnicities (2.6%), American Indian (0.1%), as well as other races/ethnicities not included within the state census (0.3%) [11].

The most frequent indication for performing radiographs was to rule out fracture following trauma to the upper extremity. Patients with a previous history of an upper extremity fracture/abnormality and/or a current fracture/abnormality were excluded. In order to perform the measurements of interest, anteroposterior (AP) radiographs including the wrist and elbow were required. Patients with incomplete or insufficient radiographs were excluded. Radiology reports and/or the attending’s office visit notes were reviewed to confirm that there was no fracture or known condition present when the radiograph was taken. Patient demographics and measurements of the amount of bowing at the proximal, middle, and distal third locations, and the location of the maximum bowed position, were recorded.

**Figure 1:**
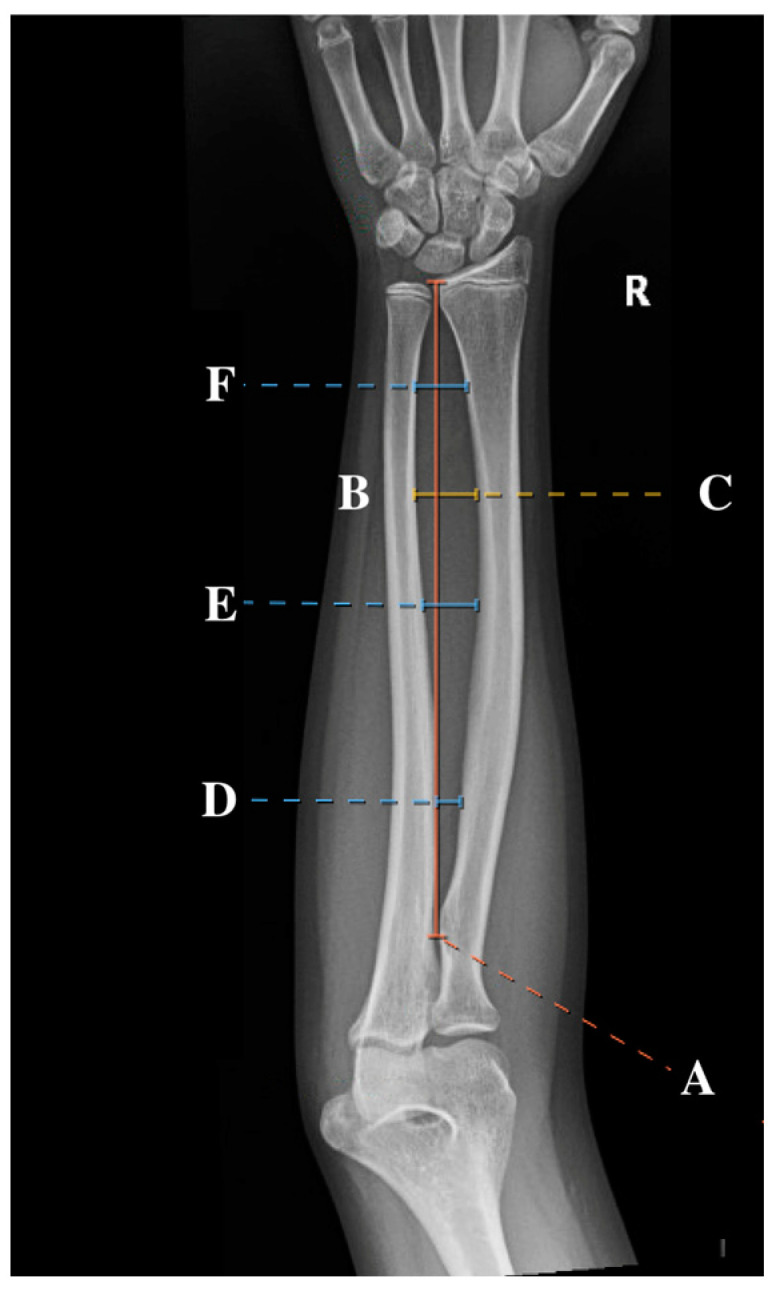
An AP radiographic view of the forearm of a 12-year-old female with the six measurements included in this study: total radial length (**A**), location of the radial bow (**B**), maximum radial bow (**C**), and proximal (**D**), middle (**E**), and distal (**F**) radial bow.

Standard AP radiographs were obtained from the IMPAX imaging system. Subsequently, the IMPAX imaging system measuring tool was used to measure the location of the maximum radial bow, as initially described by Schemitsch and Richards [4,7]. Measurements also included the total radial length (measured as the distance between the biceps tuberosity and the distal radial ulnar joint (DRUJ)), location of maximal radial bow (measured as both the distance from biceps tuberosity to the apex of the radial bow and the percentage of total radial length), and depth at maximal radial bow (measured both as the distance from the cortex of the radius at the maximum bow to the cortex of the ulna and as the percentage of total radial length). These measurements, represented as a percentage of the total radial length, were utilized to normalize the values to allow for comparison across age groups, similar to the methodology employed in previous studies [2,4,10]. In addition to these previously described measurements, the location of the proximal, middle, and distal third of the radial bow was recorded. A sample of the measurements can be found in Figure 1. All measurements were performed by four observers (SK, RL, CM, and AF) to improve generalizability to other surgeons.

Patient age was recorded as days from birth to the date of the radiograph. Age was reported as a continuous variable using mean with standard deviations, median with interquartile range, and complete range. Then, patients were categorized into age groups of less than 6 years, 6–10 years, and 11–17 years of age. Patient sex and arm laterality were also reported as categorical variables, and all were summarized with counts and percentages. Measurements were reported using means with standard deviations and ranges. There were no missing data.

Correlations between age and maximal radial bow and age and normalized values for both radial bow location and depth were explored using Pearson correlation coefficients with a confidence level of 0.95. Subgroups of male and female patients were formed, and correlations were repeated. All statistical analyses were performed using R, version 4.2.2 (R Core Team).

## 3. Results

A total of 1595 pediatric and adolescent patients with forearm radiographs were identified. Of these patients, 505 patients had no previous upper extremity fracture or condition and had adequate forearm radiographs to conduct the measurements of interest and were included in the final cohort. Of the 505 patients that met inclusion criteria, the mean age was 10.0 years ± 5.2 years (range: 0.0–16.9 years). The percentage of male patients was 51.7%, and 50.5% of radiographs were of the right forearm (Table 1).

**Figure 2:**
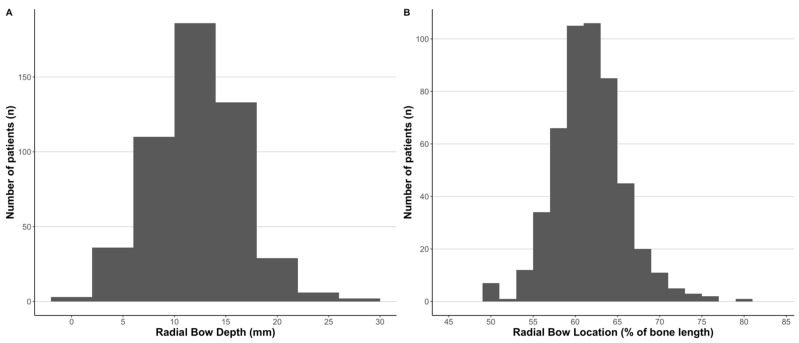
Distribution of radial bow depth in millimeters (**A**) and as a percentage of total radial length (**B**).

The total radius length from the bicipital tuberosity to the DRUJ, maximal radial bow location, and maximal radial bow depth, both measured in millimeters and represented as a percentage of the total radial length, are presented in Table 2. Among the overall cohort, the maximal radial bow was located, on average, at 61.7% ± 4.5% (range: 35.5–97.4) of the total radial length. The average maximal depth of the radial bow at its apex was 7.7% ± 1.9% (range: 2.3–15.1) of the total radial length. The breakdown of patients according to radial bow location as a percentage of the total bone length and maximal radial bow depth is presented in Figure 2.

Although the location of the maximal radial bow, measured in millimeters, increased with increasing chronological age, the location of the maximal radial bow relative to the total length of the radius did not reveal a significant association with age (r = −0.01; 95% CI: −0.10–0.08, *p* = 0.849). Additionally, the mean relative radial bow location remained consistent across age groups (Table 2). The normalized value indicates that the relative position of the maximal radial bow remains consistent as the immature skeleton develops.

**Figure 3:**
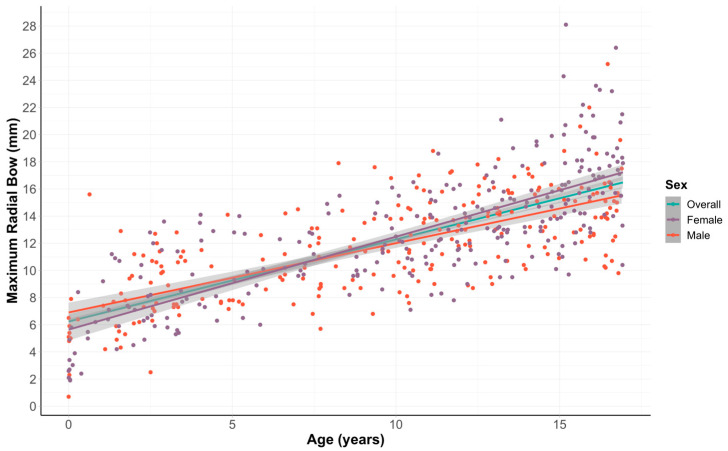
Age vs. radial bow depth, measured in millimeters.

Overall, the correlation between patient age and the depth of the maximal radial bow, measured in millimeters, was highly correlated, with r = 0.74 (95% CI: 0.70–0.78, *p* < 0.001). The correlation remained strong when evaluating subgroups by patient sex (male patients: r = 0.77 [95% CI: 0.72–0.82, *p* < 0.001], female patients: r = 0.69 [95% CI: 0.62–0.75, *p* < 0.001]) (Figure 3).

The depth of the maximal radial bow as a percentage of radial length was weakly correlated with age, with r = −0.26 (95% CI: −0.34–−0.17, *p* < 0.001) (Table 2). When normalized for radial length, the depth of the bow marginally decreased with increased age.

The proximal third, middle third, and distal third maximal radial bows similarly increased with chronological age (Table 2).

## 4. Discussion

Among the adult population, the radial bow is well characterized and utilized to guide forearm fracture care, as abnormal radial bow anatomy can cause functional limitations [4,5]. The current study investigated the radial bow among a cohort of 505 uninjured children and adolescents from ages 0 to 17 years to investigate its development and establish normative values among the pediatric and adolescent populations. To the authors’ knowledge, this study is the largest investigation of the growing radial bow within the literature. It concludes that the maximal depth of the radial bow (represented as a percentage of the total radial length) marginally decreases with increased age, but the location of the maximal radial bow (represented as a percentage of the total radial length) remains consistent as chronological age increases.

A recent study conducted by Nedder et al. utilized computed tomography (CT) scans to assess the development of the pediatric radial bow from a three-dimensional perspective [10]. The normalized maximal, distal third, and middle third radial bows were found to increase with age, while the normalized location of the radial bow and proximal third of the radial bow remain relatively constant [10]. These results suggest that the proportional shape of the radius changes throughout development, although there seems to be a “qualitative plateau at approximately 8 years of age” [10].

The normalized location of the radial bow remained consistent with increasing age in the current study, with a mean of 61.7% of the total radial bone length. These findings are comparable to that of Nedder et al., who report a mean maximal radial bow location of 56.0%, which also remained unchanged with increasing age. The results of our investigation are further validated by previous studies within the literature, which report a mean radial bow location of 50.9–60.4% [2,6,7]. Notably, Firl and Wunsch reported a mean radial bow location of 60.4% and determined that this location remained consistent with increasing age [2]. Their study was the first study to investigate the pediatric radial bow among a cohort of 100 children aged 1–15 years old. The current study builds on this initial radiographic investigation with a larger cohort and further validates that the location of the radial bow remains consistent with increasing age.

Regarding maximal radial bow depth, the current study found that, when normalized by the total radial bow length, maximal radial bow depth marginally decreased with increasing age, with a mean depth of 7.7%. These findings deviate from that of the current literature on the relationship between normalized radial bow depth and age. Nedder et al. reported a mean depth of the normalized maximum radial bow of 5.9% that increased marginally with age (r = 0.24; *p* = 0.0024); however, it seemed to plateau at age 8 [10]. Similarly, Firl and Wunsch reported a mean radial bow depth of 7.2%, which increased with age but did not exceed 10% [2]. This discrepancy may be due to the differences in sample size between the various studies. The current cohort included 505 patients, whereas the previous studies investigated 152 and 100 patients, respectively. Furthermore, there may be some inherent discrepancies as the incomplete ossification of the radius makes radiographic measurements difficult among younger patients. While CT imaging may permit more accurate measurements, CT imaging is rarely utilized clinically for acute fractures in the pediatric and adolescent populations; thus, normative values based on plain radiographic measurements are still warranted.

It is important to recognize that pediatric measurements of the radial bow have been implemented as radiographic outcomes in more recent investigations of pediatric forearm fracture treatments, akin to outcome measures used within the adult population. The utility of these measurements remains a topic of debate. Restoration of the radial bow is associated with improved functional outcomes within the adult population [4,5]. However, the anatomic restoration of the radial bow and its implication on postoperative function remains unclear within the pediatric and adolescent populations [8]. Despite this, assessment of the radial bow as an outcome measure has been utilized multiple times in studies surrounding the treatment of both pediatric and adolescent bone forearm fractures [6,7,8,9]. Celebi et al. reported no significant difference in maximal radial bow depth or location between the contralateral uninjured side and the control group for those patients that underwent flexible intramedullary nail fixation. Furthermore, functional results were largely “excellent” (96%; *n* = 26), achieving more than 90% of normal forearm rotation. Two patients in this cohort who achieved “good” functional results (80–90% of normal forearm rotation) displayed loss of radial bow depth and change in location. That said, one patient experienced a loss of radial bow depth but still achieved “excellent” functional results. Kim et al. reported significant differences among postoperative radial bow location compared to the contralateral uninjured limb; however, all patients regained complete forearm rotation [7].

Intramedullary nailing of forearm fractures has been associated with distal translation of the radial bow. However, in the aforementioned studies, there were no forearm rotational limitations [8,9,12]. A more recent investigation of the effect of radial bowing and intramedullary nailing of adult forearm fractures reported that the restoration of the radial bow does not have an impact on forearm rotation; however, abnormal bowing can impact fracture healing and grip and pinch strength [5]. However, it should be noted that the latter outcome measures have not been investigated in association with the radial bow within the pediatric population.

In addition to utilizing radial bow location and depth measurements to assess postoperative outcomes, these measurements can aid in the diagnosis of more subtle fractures. Namely greenstick fractures and plastic deformations, which are almost exclusively observed in the pediatric and adolescent populations. Both of these injuries can be difficult to diagnose due to the potential absence of a clear cortical defect, which may only be visible on either strict AP or lateral radiographic views [13,14]. Furthermore, these injuries are often initially seen by junior residents for which the subtle nature of the injury in conjunction with limited experience with pediatric trauma may lead to missed or incorrect diagnoses [13]. That said, normative values for the pediatric and adolescent radial bow may substantially aid in correctly identifying the initial diagnosis and mitigate any potential risks associated with a delayed treatment.

The current study does have several limitations. The retrospective nature of this study inherently limited the number of suitable radiographic images to investigate. Many patients who were initially identified were unable to be included in the study due to having an upper extremity fracture or inadequate radiographs. A larger cohort could be acquired from a prospective radiographic investigation of uninjured children and adolescents; however, this is often avoided to protect young patients from unnecessary radiation exposure. This study investigated length and depth measurements of the radial bow, but did not include angular measurements. Length and depth measurements were performed instead as they have been utilized to establish normative values and as outcome measures regarding the radial bow in both the pediatric and adult populations [1,2,4,5,6,7,8,9,10,12]. Furthermore, the radial bow allows for the radius to rotate around the ulna and therefore drawing an angle on a 2-dimensional AP radiograph would not accurately represent the 3-dimensional rotational arc of the bow. Additionally, the radiographic measurements included in the current study were conducted by four independent observers, which may contribute to variation between individuals; however, this methodology was utilized to improve generalizability.

## 5. Conclusions

In conclusion, the maximal depth of the radial bow marginally decreases while the location of the maximal radial bow remains consistent as chronological age increases. These measurements may be used to assess fracture reduction to ensure proper restoration of the radial bow. Furthermore, they can aid in the detection of subtle fractures of the radius, including plastic deformations or greenstick fractures.

## Figures and Tables

**Table 1:** Patient characteristics.

Characteristic	*N* = 505
Age (years)	
Mean (SD)	10.0 (5.2)
Median (IQR)	11.1 (5.3, 14.5)
Range	0.0, 16.9
Age by category, *n* (%)	
<6 Years	136 (26.9%)
6–10 Years	107 (21.2%)
11–17 Years	262 (51.9%)
Sex, *n* (%)	
Female	244 (48.3%)
Male	261 (51.7%)
Arm laterality, *n* (%) ^1^	
Right	254 (50.5%)
Left	249 (49.5%)

^1^ Percentages are out of 503 patients, as laterality was unable to be obtained from two patients.

**Table 2:** Radius bone length, bow length, and bow location by age group.

Characteristic	Overall (*n* = 505 ^1^)	<6 years (*n* = 136 ^1^)	6–10 years (*n* = 107 ^1^)	11–17 years (*n* = 262 ^1^)
Bone length (mm)	162 ± 51 (30–251)	93 ± 29 (30–207)	157 ± 20 (112–208)	200 ± 20 (111–251)
Bow location (mm)	100 ± 32 (18–159)	57 ± 18 (18–126)	98 ± 15 (64–147)	123 ± 14 (78–159)
Bow location (%)	61.7 ± 4.5 (35.5–97.4)	61.5 ± 6.0 (35.5–97.4)	62.3 ± 4.1 (50.0–73.1)	61.5 ± 3.8 (50.3–74.9)
Bow depth (mm)	12.3 ± 4.3 (0.7–28.1)	7.9 ± 3.1 (0.7–15.6)	11.6 ± 2.6 (5.7–17.9)	14.8 ± 3.4 (7.8–28.1)
Bow depth (%)	7.7 ± 1.9 (2.3–15.1)	8.6 ± 2.5 (2.3–15.1)	7.4 ± 1.5 (3.5–11.4)	7.4 ± 1.5 (4.2–13.2)
Proximal 1/3 bow (mm)	6.2 ± 2.6 (0.0–20.7)	4.6 ± 2.1 (0.0–10.0)	5.5 ± 1.7 (2.8–11.1)	7.2 ± 2.7 (1.9–20.7)
Middle 1/3 bow (mm)	10.9 ± 3.9 (0.7–26.5)	7.1 ± 2.8 (0.7–14.4)	10.1 ± 2.3 (5.6–16.1)	13.2 ± 3.3 (6.8–26.5)
Distal 1/3 bow (mm)	10.9 ± 3.8 (0.4–22.6)	7.1 ± 3.0 (0.4–14.6)	10.6 ± 2.5 (4.8–16.9)	13.0 ± 3.0 (7.2–22.6)

^1^ Values are means ± standard deviations (min–max). Bow location is presented in millimeters measured from the bicipital tuberosity of the radius and as a percentage of total bone length. Bow depth is presented in millimeters and as a percentage of bone length.

## Data Availability

All data and materials support their published claims and comply with field standards and are available for review.
